# Osteopathic Manipulation Treatment Improves Cerebro–splanchnic Oximetry in Late Preterm Infants

**DOI:** 10.3390/molecules24183221

**Published:** 2019-09-04

**Authors:** Benedetta Marinelli, Francesca Pluchinotta, Vincenzo Cozzolino, Gina Barlafante, Maria Chiara Strozzi, Eleonora Marinelli, Simone Franchini, Diego Gazzolo

**Affiliations:** 1 Department, Italian Academy of Traditional Osteopathy (AIOT), 65100 Pescara, Italy; 2 Laboratory Research, Department of Paediatric Cardiovascular Surgery, San Donato Milanese University Hospital, 20097 San Donato, Italy; 3 Department of Maternal, Fetal and Neonatal Medicine, C. Arrigo Children’s Hospital, 15121 Alessandria, Italy; 4 Neonatal Intensive Care Unit G. D’Annunzio University of Chieti, 66100 Chieti, Italy

**Keywords:** NIRS, osteopathy, late preterm, brain, splanchnic

## Abstract

**Background**: To evaluate the effectiveness/side-effects of osteopathic manipulation treatment (OMT) performed on the 7th post-natal day, on cerebro–splanchnic oximetry, tissue activation and hemodynamic redistribution in late preterm (LP) infants by using near infrared spectroscopy (NIRS). **Methods**: Observational pretest-test study consisting in a cohort of 18 LPs who received OMT on the 7th post-natal day. NIRS monitoring was performed at three different time-points: 30 min before (T0), (30 min during (T1) and 30 min after OMT (T2). We evaluated the effects of OMT on the following NIRS parameters: cerebral (c), splanchnic (s) regional oximetry (rSO2), cerebro–splanchnic fractional tissue oxygen extraction (FTOE) and hemodynamic redistribution (CSOR). **Results**: crSO2 and cFTOE significantly (*P* < 0.001) improved at T0-T2; srSO2 significantly (*P* < 0.001) decreased and sFTOE increased at T0-T1. Furthermore, srSO2 and sFTOE significantly improved at T1-T2. Finally, CSOR significantly (*P* < 0.05) increased at T0-T2. **Conclusions**: The present data show that OMT enhances cerebro–splanchnic oximetry, tissue activation and hemodynamic redistribution in the absence of any adverse clinical or laboratory pattern. The results indicate the usefulness of further randomized studies in wider populations comparing the effectiveness of OMT with standard rehabilitation programs.

## 1. Introduction

Despite recent advances in perinatal therapeutic strategies, prematurity still constitutes one of the major causes of neonatal mortality and morbidity [1]. Preterm infants are at higher risk for a variety of complications, of long stays in hospital and of long-term neurodevelopmental disabilities, often associated with higher economic and social costs [2]. In this regard, it has been reported that average days of hospitalization can range from 4 to 135 days [3] and the social costs for preterm births are estimated to be more than US$ 26.2 billion with average first-year medical costs of about US$ 32,325 [4].

In the last decade there has been increasing interest in late preterm infants (LP), who account for about 70% of total preterm births. Epidemiological data show that the risks for LP of adverse neonatal outcome are seven times more than those for term infants [5].

The post-critical neurological management of LP infants focuses on complementary and alternative treatments and on an early rehabilitation program [6]. Osteopathy (OP) is a drug-free treatment that uses a manual approach to diagnose and treat so-called somatic dysfunctions (SD). SD are commonly considered as bodily areas which manifest an altered tissue texture, a restricted range of motion, tenderness and asymmetry. These areas are characterized by a pro-inflammatory state as well as altered autonomic control [7].

OP is widely practiced in adults, especially in connection with muscular–skeletal problems and, more recently, osteopathic clinical trials have been conducted to investigate the role and impact of OP treatment in the care of preterm infants, showing a decrease in the length of hospital stays and in social costs [7]. The use of OP techniques in a perinatal setting is still a matter of debate. The term “indirect technique” refers to a gentle manipulative touch (OMT) rather than a passive touch, which has been shown to improve neonatal behavior in both animal and human models [7,8]. It has been suggested that OMT modulates autonomic nervous system functions and reduces pro-inflammatory cytokines [9,10,11,12]. However, despite these encouraging findings, data on the possible positive or side effects on neonatal brain oximetry and function are still lacking.

The purpose of the present study was to investigate whether OMT could improve or affect brain–splanchnic oximetry and function in LP infants, using near infrared spectroscopy (NIRS) monitoring before, during and after the OMT procedure.

## 2. Results

### 2.1. Main Perinatal Outcomes

Perinatal characteristics, main neonatal outcomes and standard monitoring parameters of the procedures applied to the 18 recruited infants are reported in Table 1. Gestational age (GA) and birthweight (BW) were within the 10–90° centiles for our population standards, emergency caesarean section (CS) was necessary in four out of 18 pregnant women, 11 out of 18 newborns were males and all LP admitted to the study were inborn. Maternal age at delivery was within the reference standard for our population, none of the mothers had a history of chorioamnionitis or pre-eclampsia (EPH), seven out of 18 pregnant women received antenatal steroid prophylaxis and premature rupture of membranes (PROM) occurred in three cases.

At birth all LP had an Apgar score >7 and respiratory distress syndrome (RDS) requiring surfactant administration and mechanical ventilation occurred in five out 18 LP. No LP required drug treatment for patent ductus arteriosus persistence (PDA); none of them developed intraventricular hemorrhage (IVH), early onset sepsis (EOS), necrotizing enterocolitis (NEC), retinopathy of prematurity (ROP) or bronchopulmonary dysplasia (BPD), whilst two out of 18 LP needed antibiotic treatment for late onset sepsis (LOS).

### 2.2. Monitoring Parameters

At T0 (before OMT), laboratory parameters such as venous blood pH, bilirubinemia, hemoglobin levels and hematocrit rate were within reference values. Standard monitoring parameters (heart rate, HR; respiratory rate, RR; pulsed arterial oxygen saturation, SaO_2_) at monitoring time-points were within reference values and no significant differences (*P* >0.05, for all) were found at T0-T2.

Neurological examination and cerebral ultrasound were normal in all LP admitted to the study (Table 2).

### 2.3. NIRS Parameters

**Figure 1:**
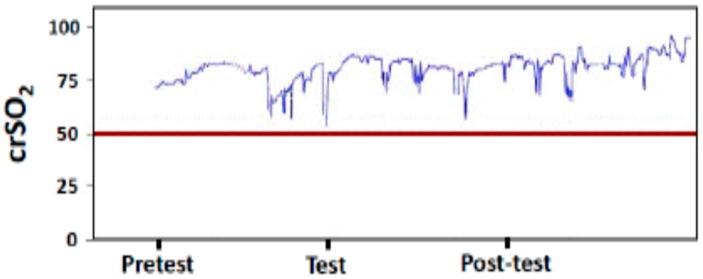
Cerebral regional oxygen saturation (crSO_2_) pattern recorded before, during and after osteopathic manipulation treatment.

NIRS parameters in cerebral district were measurable in all LP infants recruited to the study (Figure 1).

**Figure 2:**
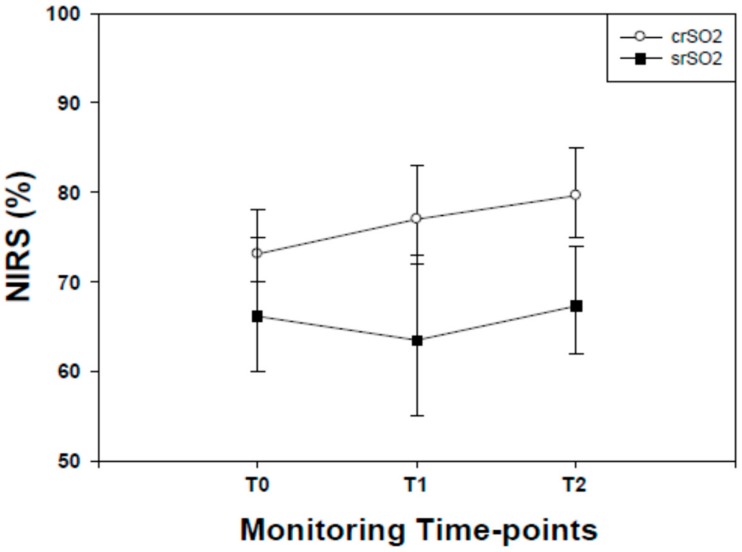
Cerebral (c) and splanchnic (s) regional oximetry (rSO_2_) values recorded in late preterm infants before (T0) during (T1) and after (T2) osteopathic manipulation treatment. Data are given as median and 25–75° centiles. crSo2 values at T0 were significantly (*P* <0.001, for both) lower than those recorded at T1 and T2. srSO2 values at T0 were significantly (*P* <0.001, for both) higher than those recorded at T1 whilst no differences (*P* <0.05) were found between T0 and T2. srSo2 values at T1 were significantly (*P* <0.001) lower than T2.

Cerebral regional oxygen saturation (crSO_2_) values started to increase from T0 to T2 (*P* <0.001, for all). Significant (*P* <0.001) differences in crSO_2_ values were found between T1 and T2 (Figure 2).

Identically, after normalizing all NIRS results with T0 values set to 0, crSO_2_ values started to increase from T0 to T2 (T0-T1 median: 3.00; 25/75° centile: 2.00/4.00; T0-T2 median: 2.00; 25/75° centile 2.00/3.00) (*P* <0.001, for all). Significant (*P* <0.001) differences in crSO_2_ values were found between T1 and T2 (T1-T2 median: 0.00; 25/75° centile −2.00/1.00).

**Figure 3:**
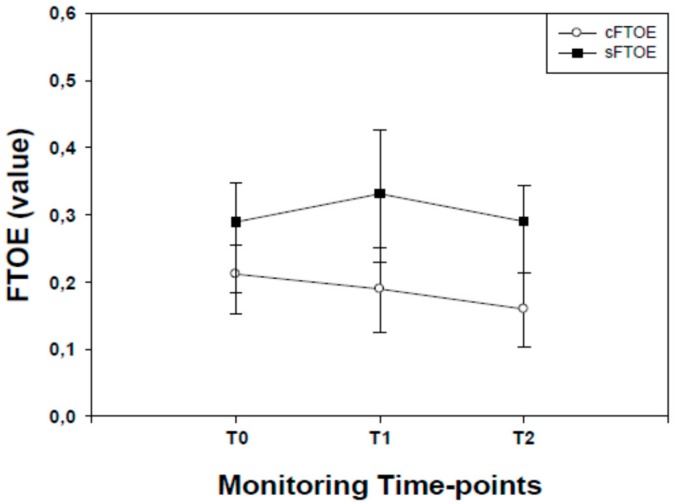
Cerebral (c) and splanchnic (s) fractional tissue oxygen extraction (FTOE) values recorded in late preterm infants before (T0) during (T1) and after (T2) osteopathic manipulation treatment. Data are given as median and 25–75° centiles. cFTOE values at T0 were significantly (*P* <0.001, for both) higher than those recorded at T1 and T2. sFTOE values at T0 were significantly (*P* <0.001, for both) lower than those recorded at T1 whilst no differences (*P* <0.05) were found between T0 and T2 time-points. sFTOE values at T1 were significantly (*P* <0.001) higher than T2.

Cerebral fractional tissue oxygen extraction (cFTOE) values started to decrease from T0 to T1, reaching their lowest point at T2 (*P* <0.001, for all). Significant (*P* <0.001) differences in cFTOE values were found between T1 and T2 (Figure 3).

**Figure 4:**
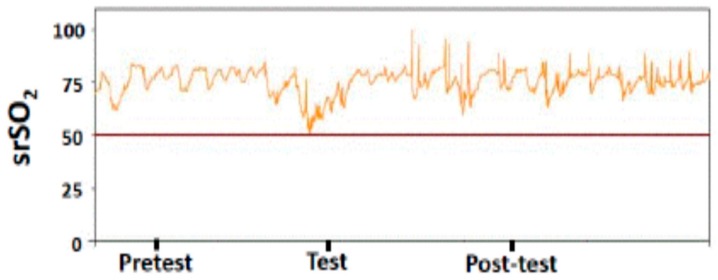
Splanchnic regional oxygen saturation (srSO_2_) pattern recorded before, during and after osteopathic manipulation treatment.

NIRS parameters in splanchnic district were measurable in all LP infants recruited to the study (Figure 4).

The pattern of splanchnic regional oxygen saturation (srSO_2_) values showed a significant (*P* <0.001) decrease in splanchnic oximetry from T0, reaching their lowest point at the end of the OMT procedure (T1).From T1 to T2, srSO_2_ values started to increase, being significantly (*P* <0.001) higher than T1 and superimposable (*P* >0.05) at T0 (Figure 2).

Identically, after normalizing all NIRS results with T0 values set to 0, srSO2 values started to decrease from T0 to T1 (T0-T1 median: −3.00; 25/75° centile: −14.00/6.00; T0-T2 median: −1.00; 25/75° centile: −10.00/8.00) (*P* <0.001, for all). Significant (*P* <0.001) differences in srSO2 values were found between T1 and T2 (T1-T2 median: 1.00; 25/75° centile: −8.00/11.00).

Splanchnic fractional tissue oxygen extraction (sFTOE) values showed a significant (*P* <0.001) increase in sFTOE from T0 reaching their peak at the end of T1. From T1 to T2 sFTOE values started to decrease being significantly (*P* <0.001) lower than T1. No differences (*P* >0.05) in sFTOE values were found between T0 and T2 (Figure 3).

**Figure 5:**
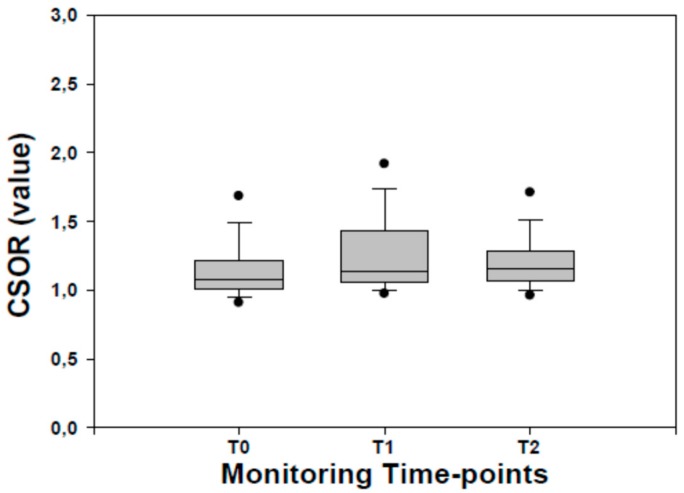
Cerebral–splanchnic ratio (CSOR) values recorded in late preterm infants before (T0) during (T1) and after (T2) osteopathic manipulation treatment. Data are given as median and 25–75° centiles.

Cerebral–splanchnic ratio (CSOR) values at TO were significantly lower (*P* <0.001, for both) than those recorded at T1 and T2. Lower (*P* <0.05) CSOR values were found between T1 and T2 (Figure 5).

## 3. Discussion

Nowadays, thanks to highly advanced medical technology in NICUs, the mortality rate for preterm infants is significantly reduced. Conversely, the incidence of disability and neurodevelopmental problems among survivors still remains high and problematic [2,5]. Based on the flat trend of morbidity rates, it has been suggested that brain injury should be considered as a complex amalgam of diseases (i.e., damage-related, maturational and trophic disturbances) rather than due to a single agent [13,14]. Alterations in neurological development/damage can occur independently of gestational age and often in the absence of evident signs of injury [15]. The less mature, healthy or sick preterm newborn may be unable or only partially able to manage environmental inputs, demonstrating over-reactive responses and poor tolerance of even minimal input. In addition to standard neuro-therapeutic strategies performed in neonatal intensive care units (NICU), an important role is played by the early introduction of individualized rehabilitation treatments [6,7,8,16]. In this regard, further progresses and new rehabilitation programs are eagerly awaited.

In the present study we showed, in a cohort of late preterm infants, that cerebral and splanchnic oximetry and tissue activation levels, evaluated by the fractional tissue oxygen extraction ratio, significantly changed during and after osteopathic manipulation treatment. Furthermore, significant hemodynamic changes in the cerebro–splanchnic regions were found during and after OMT.

To the best of our knowledge the present study constitutes the first observation in which brain oximetry and tissue activation levels were longitudinally monitored during osteopathic treatment. There are only a few observations on the potential positive effects of OMT on selected outcomes such as newborns’ hospital stays [7].

The findings of improved cerebral oximetry and tissue activation levels in LP warrant further consideration. In particular, crSO_2_ increased soon after the start of OMT and reached its highest peak 30 min after its conclusion; in parallel, cFTOE significantly decreased. This finding is noteworthy, since crSO_2_ expresses an adequate cerebral oxygenation status while cFTOE is a reliable indicator of tissue oxygen extraction, reflecting the balance between delivery and central nervous system (CNS) tissue consumption [17,18,19,20]. Thus, these findings suggest that the “over-oxygenation rate” due to the OMT procedure increased tissue metabolic activity in the CNS. The issue is of relevance bearing in mind that at this stage the major metabolic activity of the CNS concerns its growth. In an animal model and in some patients, it has been shown that the late preterm period is crucial for the development of the CNS, which reaches 65% of its total weight, and of the cerebral cortex, which reaches 53% of its total volume, while components of structural and functional brain development such as synaptogenesis and dendritic arborization reach up to 35% of their total growth [21,22,23,24]. These findings are also corroborated by a significant increase in biological fluids of the concentrations of well-established neurobiomarkers at trophic action [25,26,27]. Altogether, it is reasonable to argue that OMT improves oximetry and tissue activation levels in the CNS of newborns throughout the monitoring period and may have a beneficial effect on CNS development. The issue is also highlighted by the significant increase in CSOR values, expressing a stable preferential hemodynamic redistribution in favor of the brain. Of course, additional studies in a wider population are needed to investigate further the possible short/long term effects of OMT on CNS development and function.

In the present study, we also found that splanchnic NIRS parameters changed during and after OMT. Briefly, srSO_2_ decreased and sFTOE increased during the course of the treatment, and this was followed by a significant increase in srSO_2_ and a decrease in sFTOE from the end of treatment to the recovery period.

These findings merit further consideration. In particular, i) the significant changes in NIRS parameters during OMT seem to suggest a reduction in splanchnic oximetry with a significant change (increase in sFTOE) in the balance between delivery and splanchnic tissue consumption in favor of the former. The explanation may lie in the hemodynamic redistribution from the splanchnic to the brain area following OMT, ii) the strong recovery of NIRS parameters characterized by an increase in splanchnic oximetry and tissue activation levels (decrease in sFTOE) in the post-OMT period are suggestive, as shown by the stable improvement in NIRS parameters in the CNS, of later positive effects of OMT. This finding is also corroborated by increased CSOR ratio values (>1), as expression of a hemodynamic redistribution from the splanchnic to the brain region. Another explanation may lie in the delivery of extra blood volume by the liver, as occurs in other intrauterine conditions such as growth retardation [20,28]. Altogether, it is possible to argue that OMT may reasonably be responsible for a redistribution from the splanchnic to the brain region followed by a significantly improved splanchnic oximetry and function in the presence of a stable increased CNS oximetry and tissue activation levels. In other words, OMT seems to exert beneficial effects both on brain (early) and splanchnic (late) oximetry and function. Further studies in wider populations are needed to confirm our observation bearing in mind the variability in NIRS parameters output due to different devices and last but not least the potential disturbances in splanchnic NIRS oximetry patterns due to stool or transitional meconium that can affect recordings availability [29,30].

We recognize that the present study has several limitations. In particular: i) the small sample size, although we were able to record a considerable number of NIRS values (median: 10,500 values), thereby reducing the possibility of potential bias, ii) external light interference, which can result in photon scattering, and (iii) movement artifacts as a consequence of problems associated with the fixing of sensors [31,32].

Lastly, it should be noted that OMT and NIRS recordings were performed at least 1 h before or after feeding in order to avoid potential bias effects due to feeding regimens [33]. Yet, this possibility was avoided since each case acted as his or her own control.

In conclusion, the present results on improved CNS and splanchnic oximetry and function in late preterm infants given OMT offer additional support for the need for new individually tailored rehabilitation programs. These findings pave the way to further studies in wider populations aimed at investigating the effectiveness of OMT and standard individualized rehabilitation programs on short/long term neurological outcome.

## 4. Materials and Methods

All subjects gave their informed consent for inclusion before they participated in the study. The study was conducted in accordance with the Declaration of Helsinki, and the protocol was approved by the local Ethics Committee (n°ASO.Neonat.12.01; 2630/71).

From December 2017 to December 2018 we conducted an observational pretest–test design involving 18 LP consecutively admitted to our third-level referral centers for NICU, where they acted as their own controls. GA was determined by clinical data and by longitudinal ultrasound scan monitoring according to the nomograms of Campbell and Thoms [34] and by postnatal confirmation in agreement with Villar J. et al. [35].

The exclusion criteria were: congenital heart diseases, congenital malformations, gastrointestinal anomalies and cutaneous diseases impeding the placement of probes.

The perinatal data, neonatal characteristics and main outcomes are reported in Table 1.

### 4.1. NIRS Monitoring

Hemodynamic and oxygenation changes in the cerebral district/region were monitored using the Sen Smart X-100 NIRS device (Nonin Medical, Plymouth, MN, USA). Self-adhesive transducers that contain the light-emitted diodes and two distant Equanox Advance sensors (Nonin Medical, Plymouth, MN, USA) were fixed on the central region of the neonatal skull. crSO_2_ and srSO_2_ and SaO_2_ were calculated by the in-built software. Fractional tissue oxygen extraction values in the cerebral and splanchnic districts were assessed according to the following formula: (SaO_2_ − c(s)rSO_2_)/SaO_2_ [17,18].

We also calculated the CSOR, which is the ratio of cerebral versus splanchnic district oximetry [19], according to the following formula: crSO_2_/srSO_2_. This ratio has been found to be a valuable index of hemodynamic redistribution in chronic hypoxic infants [20].

All OMT infants were monitored on the 7th day of age at three time-points: 30 min before, 30 min during and 30 min after completion of the OMT. No differences were observed in the duration of OMT procedures (*P* >0.05).

### 4.2. Standard Monitoring Parameters and Main Outcomes

**Figure 6:**
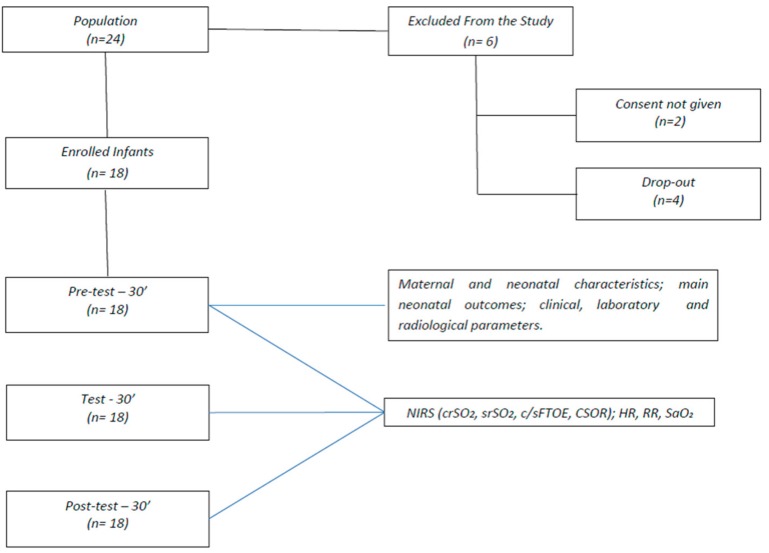
Flowchart of data collection and measurement scenarios. Abbreviations: near infrared spectroscopy, NIRS; regional oxygen saturation, rSO_2_; cerebral, c; splanchnic, s; fractional tissue oxygen extraction, FTOE; cerebro–splanchnic oxygenation ratio, CSOR; heart rate, HR; respiratory rate, RR; pulsed arterial oxygen saturation, SaO_2_.

HR and RR rates and SaO_2_ monitoring were continuously recorded by MX700 monitors (Philips, Eindhoven, The Netherlands) at 12” intervals (Figure 6).We also recorded the following main outcome measures: maternal age; the incidence of chorioamnionitis, PROM or EPH; the need of antenatal steroid prophylaxis; GA and BW; the incidence of CS, RDS, PDA, IVH, EOS, NEC, ROP, LOS or BPD.

### 4.3. Cranial Assessment

Standard cerebral ultrasonography was performed by a real-time ultrasound machine (Acuson 128SP5, Mountain View, CA, USA) using a transducer frequency emission of 3.5 MHz. Cerebral ultrasound patterns were evaluated at 24 h from birth, at 7 days and before discharge from hospital.

### 4.4. Neurological Examination

Neurological examination was performed daily according to the method of Prechtl [15]. Each infant was assigned to one of three diagnostic groups: normal, suspect, abnormal. An infant was considered to be abnormal when one or more of the following neurological syndromes were unequivocally present: (a) increased or decreased excitability (hyperexcitability syndrome, convulsion, apathy syndrome, coma); (b) increased or decreased motility (hyperkinesia, hypokinesia); (c) increased or decreased tonus (hypertonia, hypotonia); (d) asymmetries (peripheral or central); (e) defects of the central nervous system; (f) any combination of the above. When indications of the presence of a syndrome were inconclusive or if only isolated symptoms were present, e.g., mild hypotonia or only a slight tremor, the infants were classified as suspect.

### 4.5. Osteopathic Procedure

Osteopathic procedures were performed by osteopaths with experience in the neonatology field. The procedures included a structural evaluation followed by treatment. The structural evaluation was performed with the infant lying down in the open crib or incubator and was addressed to diagnose somatic dysfunctions [16]. It included rigorous and precise manual assessments of the skull, spine, pelvis, abdomen, and upper and lower limbs to locate bodily areas with an alteration in tissue, asymmetry, range of motion, and tenderness criteria [7]. The findings of that diagnostic procedure formed the basis of treatment that included the application of a selected range of manipulative techniques aimed at relieving the somatic dysfunctions. Techniques used were in line with the benchmarks on osteopathic treatment available in the medical literature and were limited to indirect techniques such as: myofascial release and balanced ligamentous/membranous tension. The whole OMT procedure on infants lasted 30 min, ten minutes for evaluation and 20 min for treatment and re-evaluation.

### 4.6. Statistical Analysis

For the calculation of sample size, we used crSO_2_ change as the main parameter. As no basic data are available for the studied population, we assumed a decrease of 0.5 SD in crSO_2_ to be clinically significant. Indeed, considering an α = 0.05 and using a two-sided test, we estimated a power of 0.80 recruiting 16 preterm infants. We therefore added 2 cases to allow for any dropout.

The sample size was calculated using nQuery Advisor (Statistical Solutions, Saugus, MA, USA), version 5.0. Main outcome measures are summarized by mean ± SD. NIRS parameters were summarized by median and interquartile ranges. Comparisons among different groups were analyzed for statistically significant differences by one way ANOVA followed by Dunn’s test. Categorical data were analyzed by means of Fisher’s test. A value of *P* <0.05 was considered statistically significant.

## 5. Conclusions

The present data offer additional support to the need of an additional and individualized rehabilitation program in the neurological care of high risk infants. Further randomized controlled trials comparing OMT with rehabilitation procedures that are considered as standard of care are, therefore, suggested.

## Figures and Tables

**Table 1:** Perinatal characteristics, main outcome measures and standard monitoring parameters recorded in late preterm infants. Data are given as mean ± SD.

	Late Preterm (N = 18)
**Maternal Characteristics**	
Maternal age (y)	32 ± 2
Chorioamnionitis (n°/tot)	0/18
Glucocorticoids (n°/tot)	7/18
PROM (n°/tot)	3/18
EPH (n°/tot)	0/10
**Neonatal Characteristics**	
GA (wks)	35 ± 1
BW (g)	1762 ± 111
CS (n°/tot)	4/18
Gender (M/F)	11/7
Outborn/Inborn (n°/tot)	0/18
Apgar 1’	8 ± 1
Apgar 5’	8 ± 1
**Main Outcomes**	
RDS (n°/tot)	5/18
Surfactant administration (n°/tot)	5/18
MV (n°/tot)	5/18
PDA (n°/tot)	0/18
IVH (n°/tot)	0/18
EOS (n°/tot)	2/18
NEC (n°/tot)	0/18
ROP (n°/tot)	0/18
LOS (n°/tot)	2/18
BPD (n°/tot)	0/10

Abbreviations: premature rupture of membranes, PROM; EPH, preeclampsia; Gestational age, GA; birthweight, BW; caesarean section, CS; respiratory distress syndrome, RDS; mechanical ventilation, MV; patent ductus arteriosus, PDA; intraventricular hemorrhage, IVH; early-onset sepsis, EOS; broncho-pulmonary dysplasia, BPD.

**Table 2:** Standard laboratory and monitoring parameters recorded before osteopathic manipulation treatment and near infrared spectroscopy performance in late preterm infants. Data are given as mean ± SD.

	Late Preterm (N = 18)
**Monitoring Parameters**	
GA (wks)	36 ± 1
BW (g)	1846 ± 265
pH	7.35 ± 0.02
pCO_2_ (mmHg)	43.9 ± 4.7
pO_2_ (mmHg)	40.1 ± 2.3
Base excess	0.9 ± 1.1
Bilirubinemia (mg/dL)	4.3 ± 1.5
Hb (g/dL)	13.9 ± 1.3
Hematocrit rate (%)	40.1 ± 2.1
HR (bpm)	146 ± 12
RR (breath pm)	56 ± 9
SaO_2_ at T0	98 ± 1
SaO_2_ at T1	98 ± 1
SaO_2_ at T2	99 ± 1
**Neurological Examination**	
Normal/suspect/abnormal	18/0/0
**Cerebral Ultrasound**	
Normal/abnormal	18/0/0

Abbreviations: gestational age, GA; weeks, wks; birthweight, BW; grams g; venous carbon dioxide partial pressure, pCO_2_; millimeter of mercury, mmHg; venous oxygen partial pressure, pO_2_; hemoglobin, Hb; heart rate, HR; respiratory rate, RR; arterial oxygen saturation, SaO_2_.
